# Impact of Precision-Guided Dosing on Clinical Decision-Making and Health Care Utilization in Inflammatory Bowel Disease: A Retrospective Pretest/Posttest Real-World Study

**DOI:** 10.1093/crocol/otaf044

**Published:** 2025-06-24

**Authors:** Ronen Arai, Adria Condino, Bincy P Abraham, Stephen B Hanauer, Udayakumar Navaneethan, Donald Lum, Syed A Hassan, Timothy Ritter, Esther A Torres, David Ziring, Harry Bray, Thierry Dervieux, Patricia Aragon Han, Terrence A Barrett

**Affiliations:** GastroHealth, Coral Springs, FL, USA; Pediatric Gastroenterology and Nutrition, FL, USA; Houston Methodist-Weill Cornell, Houston, TX, USA; Northwestern University, Feinberg School of Medicine, Chicago, IL, USA; Orlando Health, FL, USA; The Oregon Clinic GI East, OR, USA; Division of Gastroenterology and Hepatology, University of Michigan, Ann Arbor, MI, USA; GI Alliance, Southlake, TX, USA; University of Puerto Rico School of Medicine, San Juan, Puerto Rico; Cedars-Sinai Medical Center, Los Angeles, CA, USA; The Oregon Clinic GI East, OR, USA; Prometheus Laboratories, CA, USA; Prometheus Laboratories, CA, USA; Division of Digestive Diseases and Nutrition, University of Kentucky, Lexington, KY, USA

**Keywords:** precision-guided dosing, precision medicine, dose optimization, healthcare costs, therapeutic drug monitoring, pharmacokinetics, novel laboratory developed test

## Abstract

**Background:**

Precision-guided dosing (PGD) is a personalized tool that optimizes clinical decision-making in the treatment of inflammatory bowel disease (IBD) with infliximab (IFX) and its biosimilars. PGD employs nonlinear mixed-effect models using patient-specific pharmacokinetic parameters to predict infliximab trough concentrations without the need to wait until the actual trough measurement. This approach calculates patient-specific clearance (CL) and provides tailored IFX dosing and administration intervals aimed at achieving target trough levels. Implementing PGD can enhance treatment outcomes in IBD patients and may potentially reduce healthcare expenditures.

**Methods:**

We conducted a multicenter, retrospective study as a follow up to our previous clinical experience program (CEP). We aimed to evaluate the impact of PGD on clinical decision-making, patient outcomes, healthcare utilization, and expenditures. Treatment decisions included: IFX dose intensification, reduction, discontinuation, or continuation. Disease activity and healthcare resource utilization and costs in the 12 months pre- and post-test were compared. Disease activity was measured using the physician global assessment (PGA) as follows: remission (0), mild (1), moderate (2), and severe (3). Costs were calculated based on modeling pre-established literature data.

**Results:**

Analysis of data from 82 patients across 7 states and Puerto Rico showed that PGD-driven therapeutic decision making led to IFX treatment intensification (27%) or discontinuation (7%) in patients with low forecasted trough IFX concentrations, high clearance, and presence of antidrug antibody. Conversely, IFX dosage was reduced (18%) or unchanged (48%) for patients with high IFX concentrations and low clearance. There was a significant association between forecasted trough IFX levels and treatment modifications (*P* < .001). High clearance (> 0.294 L/day) was significantly associated with therapy intensification (OR 6.22, 95% CI: 2.19-19.8; *P* < .001). Following PGD, disease activity improved significantly (observed mean difference in physician global assessment: 0.378, *P* = 0.008) and healthcare resource utilization decreased. Across the entire patient population, hospitalizations decreased from 30 events pretest to 5 events posttest (*P* < .001), leading to overall cost saving.

**Conclusions:**

HCPs used the PGD test to guide treatment decisions. PGD-driven optimization of IFX therapy led to improved patient outcomes, lower healthcare utilization, and cost savings.

## Graphical Abstract

**Graphical Abstract:**
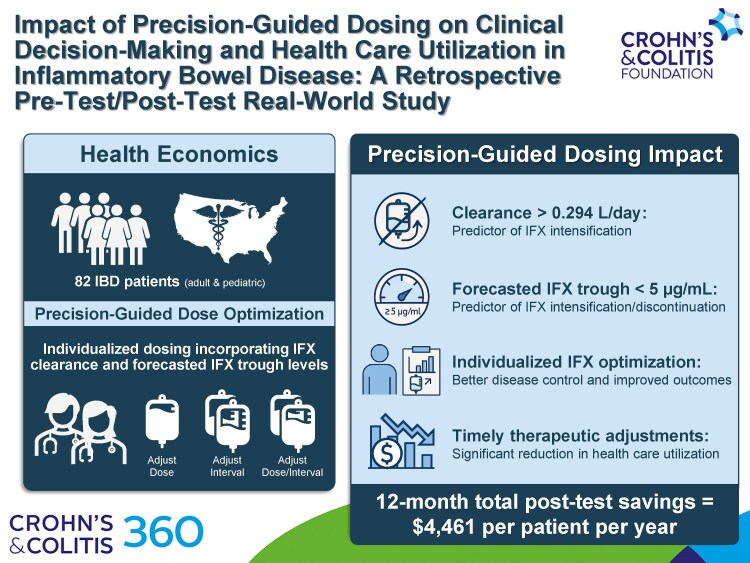

## Introduction

Inflammatory bowel disease (IBD), encompassing Crohn’s disease (CD) and ulcerative colitis (UC), is characterized by chronic relapsing inflammation leading to potential bowel damage and disability,^1^ with extraintestinal manifestations (25-50%) commonly observed.^2,3^ Treatment goals include achieving clinical remission, mucosal healing,^4^ and normalization of inflammatory biomarkers to alter underlying disease progression and improve quality of life.^4,5^

Infliximab (IFX), an anti-tumor necrosis factor alpha (TNFα) therapy, has significantly advanced IBD treatment.^6,7^ However, approximately 30% of patients do not respond initially, and approximately 50% lose response during maintenance,^8^ often due to suboptimal IFX trough concentrations and antidrug antibodies.^9^

Numerous studies confirm anti-TNFα concentrations correlate with clinical outcomes (clinical response, clinical and endoscopic remission, mucosal healing), and reduction in inflammatory biomarkers (C-reactive protein [CRP] and fecal calprotectin),^10–14^ supporting dose optimization strategies in the treat-to-target paradigm.^13,14^ However, routine dosing strategies typically rely on trough-level measurements at fixed intervals, potentially delaying dose optimization.

Precision-guided dosing (PGD), a model-informed precision dosing (MIPD) tool, addresses this limitation by utilizing forecasted IFX trough concentrations from samples collected ≥ 20 days post-infusion, corresponding to the beta (elimination) phase when drug clearance is reliably linear. A previous study^15^ validated the accuracy of forecasted trough levels, demonstrating a strong correlation with measured trough, and that forecasted concentrations above 5 µg/mL were associated with clinical and biochemical (CRP based) remission, establishing the value of PGD tools that forecast trough IFX level^15^ in expediting and optimizing informed therapeutic decision making.^15,16^ Furthermore, integrating IFX concentration with clearance further improves predictions for disease control and remission compared to either one alone.^17^

Prometheus Laboratories has developed clinically validated PGD tests for IFX and ADA or their biosimilars.^15,18^ The real-word EMPOWER (Effect on Decision-Making of Precision Optimization in Real World Evidence Research) IFX clinical experience program, including 37 health care providers (HCPs) and 275 IBD patients, demonstrated PGD significantly influenced therapeutic decisions. HCPs modified biologic therapy plans based on PGD results in 49% of cases, resulting in IFX dose modifications (41%; one-third decreased dose) and discontinuation (8%). Patients with IFX concentrations below 5 µg/mL had significantly increased odds of active disease (3-fold) and discontinuation of IFX therapy (7-fold),^19^ highlighting the impact of PGD on earlier and more precise IFX dose optimization.^19,20^

This retrospective pre-post study evaluates the long-term impact of PGD on treatment decisions, patient outcomes, healthcare resource utilization, and associated costs in a subset of EMPOWER IFX CEP patients,^19^ utilizing medical chart review and real-word data from the 12 months before and after the PGD testing to further demonstrate its clinical utility.

## Materials and methods

### Precision-guided dosing (PGD) test

The PGD test, a novel tool for IBD precision medicine, is designed to guide dose and frequency of IFX administration. The test, PredictrPK, is performed in the CLIA-certified and CAP-accredited laboratory of Prometheus Laboratories Inc., San Diego, CA. As previously described,^15,19^ it includes the determination of IFX serum concentrations (lower limit of quantification: 1.0 µg/mL), antibodies-to-IFX (ATI, lower limit of quantification: 3.1 U/mL), and albumin (g/dL). The assay results, along with IFX dose, frequency, date of last infusion, and patient weight, all provided by the HCP, are incorporated into a validated Bayesian data assimilation tool. This tool generates patient-specific PK profiles and reports IFX clearance,^17^ forecasted trough IFX concentrations, and forecasted trough IFX concentrations with alternative dosing regimens.

During the IFX induction phase, blood samples can be collected≤3 days prior to the third infusion. During the maintenance phase, samples can be collected≤3 days prior to week 14 infusion or≥20 days after any infusion (mid-cycle) up to and including trough.

### Study Design

We conducted a retrospective chart review study with a subset of patients who were part of the EMPOWER IFX CEP described in detail in Abraham et al., 2023.^19^ Briefly, adult and pediatric patients with a confirmed diagnosis of IBD (CD, UC, or indeterminate colitis [IC]) could be included in the program if they had received IFX or an IFX biosimilar for at least 14 continuous weeks (maintenance phase) with or without concomitant immunosuppressant therapy before the PGD test. Blood samples were collected any time from 20 days after an infusion to immediately prior to the subsequent infusion.

For this study, the selection of patients for medical chart reviews was at the discretion of the HCP, as long as charts with a history of 12 months before and 12 months after the test performed for the EMPOWER IFX CEP study were readily accessible. This allowed a direct comparison of the effects of optimized therapy.

Data extracted from the charts included real-world IBD-related outcomes, such as physician global assessment (PGA),^21,22^ number of clinic visits, emergency room (ER) visits, colonoscopies, magnetic resonance imaging (MRI), surgeries, hospitalizations, and length of hospital stay in the 12 months before and 12 months after the PGD test. Costs of healthcare resource utilization in the 12 months pretest and posttest were calculated based on literature data^23,24^ and adjusted to 2023 values.

### Outcome Variables and Statistical Analysis

Patients were grouped according to IFX dosage adjustments within 60 days after the PGD test. Groups were: Reduction (decrease in IFX dose and/or increase in frequency), Continuation (no change in IFX dose or frequency), Intensification (increase in IFX dose and/or decrease in frequency), and Discontinuation (switch to a different treatment, not including an IFX biosimilar). One patient was switched from IFX to an IFX biosimilar, and that patient was included in the IFX Continuation group for data analysis.

Disease activity was estimated using the PGA^21,22^ as follows: remission (0), mild (1), moderate (2), and severe (3) symptoms. Active disease was defined as a PGA score>0. Clearance (expressed in L/day) was calculated as described^17^ and values >0.294 L/day were considered as high clearance.^17^

Continuous variables were assessed for normality using the Shapiro-Wilk test. A *P*-value less than 0.05 was considered indicative of non-normality. Variables conforming to a normal distribution were reported using means and standard deviations (SDs), whereas those with non-normal distributions were reported as medians and interquartile ranges (IQRs). For univariate analyses, ANOVA tests were applied to normally distributed variables and the Kruskal-Wallis test was used for variables not meeting normality criteria. Categorical variables were reported as frequencies and percentages, associations were analyzed using the Fisher’s exact test.

Univariate and multivariate logistic regression models were used to evaluate the associations between PK predictors and changes in IFX dosage and patient outcomes, with results presented as odds ratios (OR) and 95% confidence intervals (CI). To minimize overfitting, univariate analysis was prioritized, as Prometheus’s PGD algorithm incorporates multiple clinical variables to estimate clearance and forecasted trough levels. Given the high correlation between trough concentrations and clearance, each PK predictor was tested individually in separate models. Receiver operating characteristic (ROC) analyses were performed to evaluate the predictive capability of forecasted IFX trough concentrations and IFX clearance for key clinical outcomes. The predictive accuracy of these pharmacokinetic variables was quantified using the Area Under the Curve (AUC). An AUC value greater than 0.7 was considered clinically relevant and indicative of strong predictive performance (Supplementary Figure S1).

In the comparison of healthcare resource utilization between the 12 months before and after the PGD test, the variables did not meet normality criteria. Therefore, permutation analysis was used. All pretest and posttest cases were randomly assigned without repetition to the pretest and posttest groups 10,000 times. This method allows (1) to estimate the distribution of the population; (2) to observe how the difference of means varies randomly across the shuffled dataset; and (3) to compare observed difference of mean from original data against all simulated differences in means from the shuffled data and assign a *P* value to the observed data. The *P* value represents the proportion of simulated differences of means as extreme as, or larger than, the one observed in the real data. This helps test the null hypothesis (the healthcare resource utilization is equal between pre and posttest, $H_{0}:\mu_{pretest}=\mu_{posttest}$) against the alternative (the healthcare resource utilization is lower after posttest implementation as compared to pretest, $H_{1}:\mu_{pretest}>\mu_{posttest}$).^25^ A *P* value < 0.05 was used to determine statistical significance. All statistical analysis were performed using R.^26^ Cost utilization used in the study is provided in Supplementary Tables S1 and 2.

### Ethical considerations

Because this was a chart review that posed minimal risks to patients, the Institutional Review Board (IRB) reviewing the study determined that the research was exempt from IRB oversight. No patient personal information was included in the data transmitted to Prometheus to protect patient privacy.

## Results

### Characteristics of the Clinical Practices Participating in the Chart Review

Of the 37 HCPs who participated in the EMPOWER IFX CEP, 11 performed chart reviews of a subset (5-10 per HCP) of the patients included in the program. Six HCPs were in academic centers and 5 in community practices. All HCPs in the chart review study had been in practice for more than 10 years, and all were highly experienced in treating patients with IBD.

### Baseline Patient Characteristics

Of the 275 patients enrolled in the EMPOWER IFX CEP, this study included 82 patients, of whom 9 (11%) were pediatric (<18 years). Patients resided across 7 U.S. States (*n* = 77) and Puerto Rico (*n* = 5). Twenty-four patients (29%) reported a total of 26 extraintestinal manifestations, with arthralgia being the most common, occurring in 66% of affected patients. At the time of PGD testing, 51% of patients were in clinical remission (PGA = 0), whereas 27%, 16%, and 6% had mild, moderate, and severe disease activity, respectively (data not shown). Given the small number of pediatric patients, subgroup analysis was not feasible.

For the 71 and 73 patients for whom disease activity was evaluated with objective means (endoscopy, radiography, biochemistry, and histology) pretest and posttest, respectively, the PGA was highly correlated with the objective disease activity (Spearman Correlation Coefficient [SCC] = 0.80, *P* < .001 pretest; SCC = 0.84, *P* < .001 posttest).

Overall, at the time of the PGD test, patients received a median IFX dose of 8.6 mg/kg (IQR 5.1-10.1) or 535 mg (IQR 400-787), with a normalized dosage of 7.5 mg/kg (IQR 4.3--8.8) every 6 weeks (Table 1). The median interval between doses was 8 weeks (range 6-8).

**Table 1.** Clinical and pharmacokinetic features in the entire patient population and based on IFX treatment post-PGD test

Characteristic	Overall *N* = 82 (100%)	Reduction *N* = 15 (18%)	Continuation *N* = 39 (48%)	Intensification *N* = 22 (27%)	Discontinuation *N* = 6 (7%)	*P value*
**Demographics**						
Age, years	40 (20-51)	30 (16-50)	41 (23-51)	40 (23-52)	44 (26-46)	.45
Sex, male	46 (56)	8 (53)	21 (54)	11 (50)	6 (100)	.15
Race, white	66 (80)	12 (80)	34 (87)	17 (77)	3 (50)	.10
**Disease history and therapies**						
Diagnosis: CD	51 (62)	8 (53)	23 (59)	17 (77)	3 (50)	.30
IC	1 (1.2)	1 (6.7)	0 (0)	0 (0)	0 (0)	
UC	30 (37)	6 (40)	16 (41)	5 (23)	3 (50)	
Disease duration, years	8 (4-19)	8 (2-11)	10 (6-20)	7 (3-19)	17 (2-21)	.68
Concurrent medications	29 (35)	6 (40)	13 (33)	7 (32)	3 (50)	.83
Exposed to prior biologics	34 (41)	4 (27)	18 (46)	10 (45)	2 (33)	.59
**Montreal Classification for CD**						
Location: L1 ileal	14 (27)	1 (11)	9 (39)	4 (24)	0 (0)	.07
L2 colonic	6 (12)	2 (33)	0 (0)	3 (18)	1 (33)	
L3 ileocolonic	31 (61)	5 (56)	14 (61)	10 (59)	2 (67)	
L4 upper disease	1 (2)	0 (0)	1 (4)	0 (0)	0 (0)	
Behavior: B1 non-stricturing, non-penetrating	19 (38)	5 (63)	11 (48)	2 (13)	1 (33)	**.04**
B2 stricturing	12 (24)	0 (0)	7 (30)	4 (25)	1 (33)	
B3 penetrating	19 (38)	3 (38)	5 (22)	10 (63)	1 (33)	
*P* perianal disease	18 (35)	2 (25)	9 (39)	5 (29)	2 (67)	
** Montreal Classification for UC and IC**						
Extent: E2 left sided	10 (32)	3 (43)	6 (38)	0 (0)	1 (33)	.40
E3 extensive	21 (68)	4 (57)	10 (63)	5 (100)	2 (67)	
Severity S0 remission	15 (58)	4 (80)	9 (64)	1 (20)	1 (50)	.34
S1 mild	1 (3.8)	0 (0)	0 (0)	1 (20)	0 (0)	
S2 moderate	5 (19)	1 (20)	2 (14)	2 (40)	0 (0)	
S3 severe	5 (19)	0 (0)	3 (21)	1 (20)	1 (50)	
**IFX pharmacokinetics**						
Normalized q6 IFX dose*, mg/kg	7.5 (4.3-8.8)	8.5 (7.8-12.9)	7.6 (4.3-8.9)	5.0 (4.1-7.4)	6.5 (4.2-9.4)	**.002**
Interdose interval, days	56 (42-56)	42 (35-56)	56 (42-56)	56 (56-56)	56 (46-56)	**.04**
Forecasted trough, µg/mL	13 (6-20)	24 (19-29)	14 (9-20)	4 (3-6)	4 (2-8)	**<.001**
Clearance, L/day	0.273 (0.224-0.344)	0.230 (0.180-0.258)	0.238 (0.199-0.313)	0.342 (0.293-0.368)	0.344 (0.295-0.421)	**<.001**
ATI, > 3.1 U/mL	10 (12)	0 (0)	1 (2.6)	7 (32)	2 (33)	**<.001**
Mean albumin (SD), g/dL	3.87 (0.43)	3.99 (0.42)	3.88 (0.44)	3.80 (0.44)	3.73 (0.40)	.49
**Healthcare management processes**						
Prior authorization	19 (23)	1 (7)	0 (0)	17 (77)	1 (17)	**<.001**
Other IFX levels & ATI Test Ordered	6 (7.3)	1 (6.7)	1 (2.6)	4 (18)	0 (0)	.16
Other changes in concurrent medication	7 (9)	1 (7)	4 (10)	2 (9)	0 (0)	.71

Of the 82 patients included, 9 (11%) were pediatric (< 18 years). Due to the small sample size, a pediatric subgroup analysis was not performed. *IFX dosing normalized to a 6-week (q6) infusion interval to facilitate interpretation. Values are presented as *n* (%) or median (IQR) unless specified. Statistical analysis consisted of Kruskal–Walli’s rank sum test for continuous variables and Fisher’s exact test for categorical variables; *P* values < .05 are bolded. ATI: anti-infliximab antibodies; CD: Crohn’s disease; IC: indeterminate colitis; IFX: infliximab or biosimilars; IQR: interquartile range; UC: ulcerative colitis.

The test was ordered for proactive (62%) or reactive monitoring (34%) or because of adverse reactions (3.7%), similarly to the entire cohort in the EMPOWER IFX CEP. Samples were collected at trough (*n* = 42, 51%) or midcycle (*n* = 40, 49%) (data not shown).

### Impact of the PGD test on treatment decisions and disease activity

To evaluate the impact of the PGD test on outcomes, patients were grouped based on whether IFX treatment was changed, as described under Methods. Detailed quartile analysis of forecasted IFX trough concentrations and drug clearance is provided in Supplementary Tables 3 and 4. For most patients (*n* = 39, 48%), IFX therapy was continued unchanged, confirming appropriate dosing, while it was reduced for 15 patients (18%), intensified for 22 (27%), and discontinued for 6 (7%). Demographic characteristics and Montreal classification criteria of the entire population and the 4 groups are reported in Table 1.

HCPs decided to reduce or continue IFX therapy for the patients with the highest forecasted trough IFX concentrations (median 24 and 14 µg/mL, respectively), while IFX was intensified or discontinued for the patients with the lowest IFX concentrations (median 4 µg/mL for both) (Table 1). The Intensification and Discontinuation groups presented with the highest median clearance, exceeding the value of 0.294 g/L that has been shown to be associated with worse disease control.^17^ No patients in the Reduction group and 1 patient (2.6%) in the Continuation group had measurable ATI, while 7 of 22 (32%) and 2 of 6 (33%) patients in the Intensification and Discontinuation groups, respectively, had ATI (Table 1). Differences among the groups in IFX dosage, forecasted IFX concentrations, clearance, and presence of ATI were statistically significant, whereas albumin concentrations were similar in the 4 groups (Table 1). Prior authorization was required mainly to increase IFX therapy in the Intensification group (17 of 22 patients, 77%, *P* < .001) (Table 1).

The therapy changes instituted following the reporting of PGD to ordering HCP led to disease activity improvement 9-12 months later without the need to perform additional TDM. In fact, after the PGD test, TDM was performed for only 7.3% of patients, with similar percentages in the 4 patient groups (2.6 to 18%, *P* = .16) (Table 1). The majority of patients in the reduction group had low disease activity pretest and also posttest (PGA = 0 for 80% and 87% of patients, respectively (Chi-squared *P* = .036). Similarly, patients in the continuation group were mainly in remission pretest (56%) and this percentage increased to 85% posttest (McNemar’s *P* = .003); in the Intensification group, PGA was 0 for 27% of patients pretest and 64% posttest (McNemar’s *P* = 0.027)). In addition, 27.5% of patients in the Intensification group had PGA of 2 and 3 pretest, and this percentage was reduced to 13.6% after intensification of IFX therapy posttest. When the PGA of the entire cohort was analyzed by permutation test, the PGA posttest was lower than pretest with an observed mean difference of 0.378 (*P* = .008) (Table 3).

**Table 3.** IBD-related healthcare utilization in the entire patient population

Health care utilization category			Mean (SD)		Difference in means	*P* value
	Pretest	Posttest	Pretest	Posttest		
Hospitalization stays, *n*	30	5	0.37 (0.76)	0.06 (0.24)	−0.305	**<.001**
Duration, days	142	16	1.7 (4.4)	0.20 (0.91)	−1.540	**<.001**
Office visits, *n*	257	240	3.13 (2.54)	2.93 (2.43)	−0.207	.612
ER visits, *n*	9	7	0.11 (0.31)	0.09 (0.32)	−0.024	.806
Urgent care visits, *n*	0	1	0 (0)	0.01 (0.11)	0.012	1.000
Surgeries, *n*	12	4	0.15 (0.45)	0.05 (0.22)	−0.098	.131
All imaging, *n*	90	64	1.10 (1.32)	0.78 (1.03)	−0.317	.098
Colonoscopies	41	42	0.50 (0.57)	0.51 (0.61)	0.012	1.000
Sigmoidoscopies	5	2	0.06 (0.24)	0.02 (0.16)	−0.037	.451
MRI	14	5	0.17 (0.38)	0.06 (0.24)	−0.110	**.050**
Other	30	15	0.37 (1.05)	0.18 (0.69)	−0.183	.226
Laboratory tests, *n*	696	580	8 (8)	7.1 (6.1)	−1.415	.194
Albumin	361	294	4.4 (4.1)	3.58 (2.66)	−0.817	.137
C−reactive protein	274	236	3.3 (3.4)	2.88 (2.93)	−0.463	.371
Fecal calprotectin	61	50	0.74 (1.14)	0.61 (1.14)	−0.134	.497
Referrals, *n*	39	34	0.48 (1.06)	0.41 (0.77)	−0.061	.735
Disease activity (PGA), remission/mild/moderate/severe	42/22/13/5	62/11/6/3	0.77 (0.93)	0.39 (0.78)	−0.378	**.008**

Difference was calculated as the average of posttest values minus the average of pretest values. Thus, negative values represent a decrease posttest compared to pretest. Statistical analysis consisted of Permutation test. ER: Emergency room; MRI: magnetic resonance imaging; PGA: physician global assessment; *P* values ≤ .05 are bolded.

**Figure 1.**
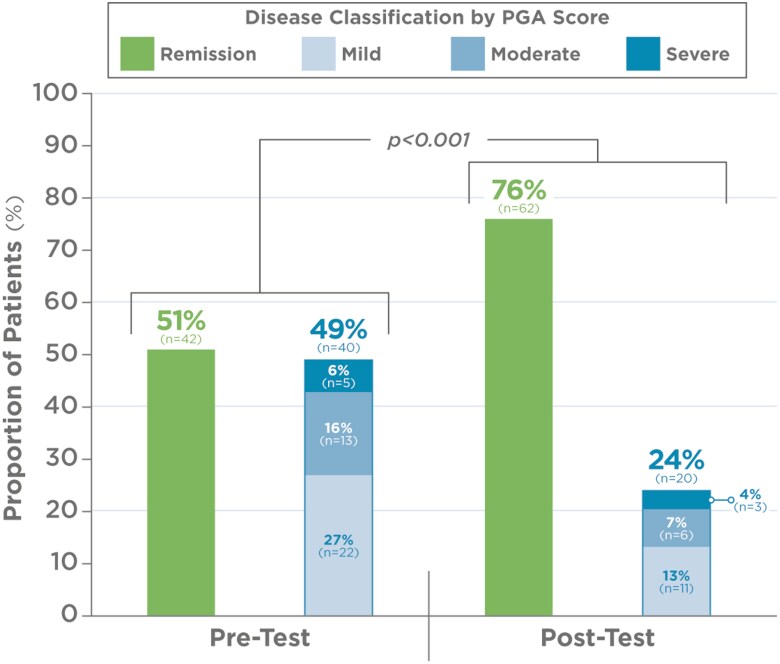
Disease activity before (pre) and ~12 months after (post) PGD test.

**Figure 2.**
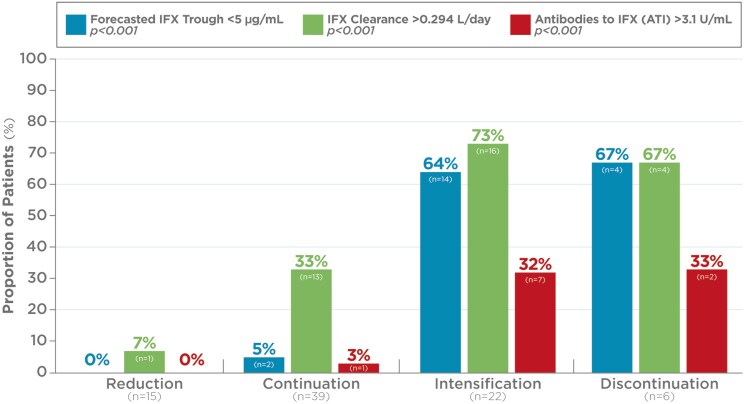
Percentage of patients with low forecasted trough IFX concentration < 5 µg/mL, accelerated clearance > 0.294 L/day, and detectable levels of anti-drug antibodies (> 3.1 U/mL) in the 4 patient groups (Reduction, Continuation, Intensification, and Discontinuation). The numbers above the bars represent percentages, while the number inside the bars indicate the number of patients in each group presenting with forecasted trough IFX concentration < 5 µg/mL, clearance > 0.294 L/day, and detectable levels of anti-drug antibodies (ATI > 3.1 U/mL)

Statistical analysis using Chi-squared test showed significant differences in PGA across the 0, 1, 2, and 3 categories when compared before and after the PGD test (*P* < .001). Furthermore, McNemar test comparing groups with active disease (PGA > 0) and remission (PGA = 0) at pretest and posttest was significant (*P* < .001) (Figure 1). The percentage of patients with forecasted trough IFX concentration < 5 µg/mL, clearance > 0.294 L/day, and detectable levels of anti-drug antibodies (> 3.1 U/mL) were statistically significant among the 4 patient groups (Figure 2).

Univariate logistic regression evaluated the association between PK parameters as independent predictors for IFX treatment decisions and active disease (PGA > 0). Low forecasted trough IFX concentrations (< 5 µg/mL) and high clearance (> 0.294 L/day) were significantly associated with therapy intensification (OR 15.8, 95% CI: 4.95-57.0, *P* < 0.001 and OR 6.22, 95% CI: 2.19-19.8, *P* < 0.001, respectively). Conversely, high forecasted trough IFX concentrations (> 10 µg/mL) were associated with therapy continuation (OR 2.59, 95% CI: 1.06-6.56, *P* = .037, respectively). Concentrations > 15 µg/mL were associated with therapy reduction (OR 12.7, 95% CI: 3.55-61.3, *P* < 0.001). Concentrations ≤ 5 µg/mL were associated with active disease, although the value did not reach statistical significance (Table 2).

**Table 2.** Univariate logistic regression analysis of pharmacokinetic predictors of IBD outcomes

Characteristic	OR (95% CI)	*P* value
Forecasted trough IFX < 5 μg/mL		
IFX therapy intensification	15.80 (4.95 to 57.0)	**<.001**
IFX therapy discontinuation	7.50 (1.34 to 57.7)	**.02**
Active disease	2.78 (0.91 to 8.35)	.07
Hospitalizations	0.76 (0.04 to 5.56)	.81
Forecasted trough IFX > 10 μg/mL		
IFX therapy continuation	2.59 (1.06 to 6.56)	**.04**
Remission	1.36 (0.38 to 6.48)	.20
Forecasted trough IFX > 15 μg/mL		
IFX therapy reduction	12.70 (3.55 to 61.3)	**<.001**
Remission	1.77 (0.60 to 6.02)	.31
IFX Clearance > 0.294 L/day		
IFX therapy intensification	6.22 (2.19 to 19.8)	**<.001**
IFX therapy discontinuation	3.07 (0.56 to 23.1)	.20
Active disease	2.07 (0.75 to 5.88)	.16
Hospitalizations	0.00 (0-$9.06\times 10^{77}$ *)	N/A

IFX clearance cutoff ≤ 0.294 L/day was previously established to best differentiate IBD patients at higher likelihood of achieving and sustaining CRP-based clinical remission.^17^ IFX: Infliximab or biosimilars; CI: confidence intervals; IBD: inflammatory bowel disease; *N*/A: not applicable; OR: odds ratio; *P* values < 0.05 are bolded. * OR = 0 and the wide confidence interval suggests model or data limitations, such as a small sample size or rare events.

For the significant predictors identified in univariate analysis (Table 2), multivariate logistic regression was performed (Supplementary Table S5). Forecasted IFX trough < 5 μg/mL remained significantly associated with IFX therapy intensification (OR 26.7, 95% CI 4.1-260.8, *P* = .002), and forecasted trough > 15 μg/mL with reduction (OR 14.0, 95% CI 2.7-101.9, *P* = .003). Clearance > 0.294 L/day was also associated with therapy intensification (OR 12.6, 95% CI 2.5-99.1, *P* = .006). A trend toward an association between forecasted trough < 5 μg/mL and discontinuation was observed (*P* = .09), as well as active disease and discontinuation (*P* = .05). Interestingly, anti-drug antibody positivity was found to be independently associated with lower odds of therapy continuation (OR 0.06, 95% CI 0.002-0.62, *P* = .04).

Receiver operating characteristic (ROC) analyses evaluated the predictive capability of forecasted IFX trough concentrations and clearance for key clinical outcomes, quantified by the area under the curve (AUC). An AUC greater than 0.7 was considered clinically relevant, indicating strong predictive performance. Forecasted IFX trough significantly predicted IFX therapy intensification (AUC = 0.857, *P* = .03) and therapy reduction (AUC = 0.905, *P* = .002). Clearance was significantly predictive of therapy reduction (AUC = 0.753, *P* = .03) and showed trends toward predicting continuation and active disease (AUC range: 0.736-0.782), though these did not reach statistical significance. Detailed ROC curves and summary statistics are provided in Supplementary Figure S1.

### Impact of the PGD test on IBD-related healthcare utilization

The test had a significant impact on healthcare utilization, with lower hospitalizations (30 vs. 5, permutation test *P* < .001) and shortened length of hospital stays (mean difference 1.54 days, *P* < .001) in the 12 months posttest compared to the 12 months pretest. There were also fewer laboratory tests, imaging procedures, surgeries, emergency room (ER) visits, and office visits posttest compared to pretest, although these values did not reach statistical significance (Table 3).

### Impact of the PGD test on IBD-related healthcare costs.

The economic impact on IBD-related healthcare resource utilization for the entire cohort after the implementation of PGD was estimated to result in total savings of $365,763 in the 12 months following testing. This corresponds to an average cost decrease of approximately $4,461 per patient per year.

Table 4 presents a detailed summary of the healthcare resources utilization raw data, outlining the differences in critical variables observed before and after the PGD test. The data is categorized based on the changes in IFX dosage across the different groups and used for the proper economic calculations crossing over the cost reported in Supplementary Table S1.^23,24^

**Table 4.** IBD-related healthcare utilization and economic impact by group

	Overall (*n* = 82)	Reduction (*n* = 15)	Continuation (*n* = 39)	Intensification (*n* = 22)	Discontinuation (*n* = 6)
∆ Total IFX, mg/infusion*	+300	-3,348	0	+6,648	-3,000
∆ Total surgeries, *n*	−8	0	-1	-6	-1
∆ Hospitalization stays, *n*	−25	-−	-10	-9	-2
∆ Duration, days	−126	-15	-−5	-62	-−
∆ Office visits, n**	−17	-12	-7	-2	+−
∆ ER visits, *n*	−2	0	−1	−1	0
Economic impact	−$365,763	−$212,102	−$173,243	+$65,283	−$45,701

Data is presented as difference (∆) posttest minus pretest. Thus, negative values represent a decrease posttest compared to pretest. ER: emergency room; IFX: infliximab or biosimilars. *Total IFX mg every 6 weeks based on individual IFX dose. **Office visits refer to any visit to the IBD specialist.

IFX dose decreased by a total of 3,348 mg every 6 weeks in the reduction group. Notwithstanding the reduction, this group experienced fewer hospitalizations (4 events), a reduction in hospitalization duration by 15 days, and 12 fewer IBD-related office visits. These changes resulted in considerable cost savings, with the most substantial contributions coming from the reduction in IFX treatment costs, totaling approximately $147,965, and a decrease in hospitalization-related costs of $63,327. Overall, the total cost savings for this group amounted to $212,102 in the year after the test.

Despite the stable IFX dosage, the continuation group had lower healthcare utilization, including 10 fewer hospitalization events, a decrease of 45 hospitalization days, and 7 fewer IBD-related office visits. Additionally, there was a reduction of 1 surgery and 1 ER visit. Therefore, cost savings were substantial, particularly in hospitalization-related expenses, which amounted to $158,317. In total, the cost savings for the Continuation group reached $173,243 in the year after the test.

Not surprisingly, the Intensification group underwent a significant increase in IFX dosage, for an additional 6,648 mg every 6 weeks, which amounted to approximately $293,895 in the 12 months following testing. These costs were partially offset by savings from reduced hospitalizations (9 events), hospitalization days (62 days), and surgeries (6 fewer), but overall, there was a net increase of $65,283 for this group in the year after the test.

The 6 patients in the Discontinuation group were switched within 6 weeks after the PGD test to adalimumab, upadacitinib, vedolizumab, ozanimod (*n* = 1 each), and ustekinumab (*n* = 2) (Supplementary Table S2). The costs of these therapies^27^ are not included in the economic impact because the duration of treatment is unknown. However, this group experienced reductions in hospitalizations (2) and hospitalization days (4), resulting in a $45,701 decrease in costs in the year after the test.

## Discussion

The PGD test provides forecasted IFX trough levels, estimated clearance, and alternative dosing strategies,^18,21^ allowing individualized treatment plans beyond traditional TDM. However, whether these adjustments improve long-term outcomes or reduce healthcare costs remained unclear. This pretest-posttest study, following the EMPOWER-IFX CEP evaluated the 12-month impact of PGD-guided IFX adjustments on clinical outcomes and healthcare resource utilization.

Baseline characteristics were similar across groups, except for disease activity, highest in patients later requiring IFX intensification or discontinuation. The PGD test enhanced patient outcomes, with an increase in the percentage of patients in remission (PGA = 0) and a decrease in the percentage of patients with more active disease. Patients undergoing IFX intensification and discontinuation had lower forecasted IFX trough concentrations, higher clearance, and a higher ATI incidence, consistent with known associations between low IFX trough levels, antibodies, and increased clearance.

Clearance, reflecting drug consumption rate, is crucial in optimizing anti-TNFα therapy. Previous studies demonstrated that IFX concentration combined with clearance predicts clinical and CRP-based remission better than either parameter alone,^17^ reinforcing the clinical relevance of clearance. Our findings confirm that high clearance (>0.294 L/day) aligns with therapy intensification or discontinuation, while lower clearance associates with unchanged or reduced IFX dosing.

IFX dose intensification, associated with low forecasted IFX concentrations (< 5 µg/mL), increased medication costs, but reduced episodes of hospitalizations, hospitalization duration, and surgeries. These data are consistent with the improved clinical response, remission, mucosal healing, and lower CRP and fecal calprotectin that have been shown in other studies that evaluated the association between concentrations of anti-TNFα and clinical response.^10–14^

IFX discontinuation due to low forecasted IFX serum concentrations and high clearance led to estimated pharmacological spending ($636,357/year), although this cost is not included in the overall economic analysis due to unknown treatment duration. Benefits in this group included improved clinical outcomes: reduced disease activity, hospitalizations, and hospitalization duration.

Patients maintaining their IFX regimen post-PGD (48%) had low clearance and optimal forecasted IFX levels (5-10 µg/mL or >10 µg/mL), showing improved outcomes and decreased healthcare costs driven by fewer hospitalizations and office visits. PGD provided confidence to HCPs that current dosing was adequate, positively influencing patient management, and therefore disease control and healthcare utilization.

Patients with reduced IFX dose (18%) had low clearance and high forecasted IFX trough (>15 µg/mL), allowing therapy reduction without negative outcomes. This group demonstrated improved clinical status post-PGD, leading to fewer office visits and hospitalizations, with substantial cost savings ($212,102/year).

Overall, PGD significantly improved clinical outcomes that resulted in a marked reduction in hospitalizations (30 pretest vs. 5 posttest, *P* = .001) and hospitalization duration. Notably, patients in this study were hospitalized 30 times in the 12 months before the PGD test. The large number of pretest hospitalizations highlights the urgency for the timely optimization of patient care.

The reduction in office visits, ER visits and surgeries were not statistically significance, possibly due to the small sample size and short follow up period highlighting the complex nature of therapy optimization and its impacts on healthcare utilization since certain aspects of healthcare utilization remain constant or are more difficult to capture adequately with a one-year follow-up.

For most patients (92.7%), TDM was not repeated after the PGD test, indicating the observed improved outcomes were not influenced by additional TDM tests. The reduced healthcare resource use after PGD likely also lowered indirect costs and improved patient satisfaction, although this could not be assessed due to study limitations.

This study demonstrates that PGD-driven individualized therapy optimization improves patient outcomes, leading to healthcare cost savings ($4,461 per patient). These findings support guidelines^28^ advocating personalized therapeutic approaches despite insurance and pharmacy benefit management resistance.^28^ Additionally, our results confirm that treatment adjustments based on forecasted IFX trough levels and clearance rather than trough concentrations alone are feasible and beneficial.^16,17,29^ These results can inform protocols aimed at cost-effective IFX management strategies. PGD can be performed periodically during IFX treatment to effectively manage the dose and interval of administration.

Study limitations include its retrospective design, small sample size, and subjective PGA disease activity assessment, although objective measures correlated significantly. Longer-term studies are warranted to fully capture outcomes such as surgery rates. Further research is needed to address healthcare access barriers imposed by insurance requirements, prior authorizations, step therapy policies, and gaps in disease monitoring and PK assessment.^28^

## Conclusion

The PGD test reports IFX concentrations, anti-drug antibody levels, clearance, and forecasted IFX concentrations at alternative IFX doses and intervals for personalized treatment of patients with IBD on IFX or biosimilars. High clearance and low forecasted trough drug concentrations were associated with IFX intensification or discontinuation, while IFX treatment was reduced or continued unchanged in the presence of low clearance and high forecasted trough drug concentrations. The long-term follow-up in this study allowed demonstration of enhanced disease control, lower healthcare utilizations, and costs following the PGD test.

## Supplementary Material

otaf044_suppl_Supplementary_Tables_S1-S5

otaf044_suppl_Supplementary_Figure_S1

## Data Availability

Data will be shared upon reasonable request to the corresponding author.
