# Supplementary material for: Impact of Precision-Guided Dosing on Clinical Decision-Making and Health Care Utilization in Inflammatory Bowel Disease: A Retrospective Pretest/Posttest Real-World Study
Source: Crohns Colitis 360. 2025 Jun 24;7(3):otaf044. doi: 10.1093/crocol/otaf044 (PMC12305531; doi:10.1093/crocol/otaf044)
Supplement: otaf044_suppl_Supplementary_Tables_S1-S5 [file otaf044_suppl_supplementary_tables_s1-s5.docx]

**Impact of Precision-Guided Dosing on Clinical Decision-Making and Health Care Utilization in Inflammatory Bowel Disease: A Retrospective Pretest/Posttest Real-World Study.**

**Supplementary material.**

**Quartile Analysis Results**

Patients were divided into quartiles based on forecasted IFX trough concentrations (Supplementary Table 3). Those in the lowest trough quartile (0.18–5.4 µg/mL) experienced notably higher rates of IFX therapy intensification (71%), discontinuation (19%), and active disease (38%). In contrast, patients in the highest quartile (20.3–52.7 µg/mL) had no cases of intensification or discontinuation, with therapy commonly continued unchanged (50%) or significantly reduced (50%). Fisher’s exact tests confirmed significant associations between trough quartiles and IFX therapy continuation (p < 0.001), therapy reduction (p = 0.030), and therapy intensification (p = 0.002).

Similarly, when patients were categorized based on IFX clearance quartiles (Supplementary Table 4), those in the highest clearance quartile (0.352–0.923 L/day) had substantially higher rates of therapy intensification (50%) and active disease (45%) compared to patients in the lowest clearance quartile (0.044–0.224 L/day), where intensification was rare (5%), discontinuation was absent, and active disease was less frequent (19%). Fisher’s exact tests showed significant associations between clearance quartiles and both therapy intensification (p = 0.002) and therapy reduction (p = 0.030), highlighting meaningful clinical differences linked to drug clearance.

**REFERENCES**

1. Xu F, Liu Y, Wheaton AG, Rabarison KM, Croft JB. Trends and Factors Associated with Hospitalization Costs for Inflammatory Bowel Disease in the United States. *Appl Health Econ Health Policy.* 2019;17(1):77-91.

2. Negoescu DM, Enns EA, Swanhorst B, et al. Proactive Vs Reactive Therapeutic Drug Monitoring of Infliximab in Crohn's Disease: A Cost-Effectiveness Analysis in a Simulated Cohort. *Inflamm Bowel Dis.* 2020;26(1):103-111.

3. Jairath V, Cohen RD, Loftus EV, Jr., Candela N, Lasch K, Schultz BG. Evaluating cost per remission and cost of serious adverse events of advanced therapies for ulcerative colitis. *BMC Gastroenterol.* 2022;22(1):501.

**Supplementary Table 1. Healthcare resources costs.**

| **Variable** | **Average Cost (USD)** | **Adjusted to Inflation 2023**  **(USD)*** | **Source** |
| --- | --- | --- | --- |
| IFX Price (per mg) | 5.1 | 5.1 | Average WAC price in the US. (<https://www.drugs.com/price-guide/infliximab>) |
| Hospitalization Related IBD (per discharge) | 12,378.5 | 15,831.7 | Xu et al. (2018)^1^ |
| Surgeries Related IBD (one time) | 11,613.7 | 14,407.7 | Negoescu et al. (2019)^2^ |
| ER Visits / Clinician Fee (one time) | 118.8 | 146,4 | Negoescu et al. (2019)^2^ |
| Office Visits (8-week period) | 54.8 | 67,5 | Negoescu et al. (2019)^2^ |

ER: emergency room; IBD: inflammatory bowel disease; IFX: infliximab or biosimilars; US-HCUP: United Stated Healthcare Cost and Utilization Project; USD: United State Dollar; WAC: Wholesale Acquisition Cost. *Calculated by <https://www.bls.gov/data/>

**Supplementary Table 2. Treatment cost after IFX discontinuation.**

| **Discontinuation Patients** | **Treatments after IFX discontinuation** | **Cost per year (USD)** | **Total cost per year (USD)** | **Sources** |
| --- | --- | --- | --- | --- |
| 1 | Adalimumab | 89,523 | 89,523 | Jairath et al. (2022)^3^ |
| 1 | Upadacitinib | 77,175 | 77,175 | <https://www.rinvoq.com/cost> |
| 2 | Ustekinumab | 150,422 | 300,844 | Jairath et al. (2022)^3^ |
| 1 | Vedolizumab | 66,777 | 66,777 | Jairath et al. (2022)^3^ |
| 1 | Ozanimod | 102,038 | 102,038 | <https://www.zeposia.com/ulcerative-colitis> |

IFX: infliximab or biosimilars; USD: United State Dollar.

**Supplemental Table 3: Clinical outcomes stratified by IFX forecasted trough level quartiles**

| **Quartile**  **(sample n)** | **Trough range (µg/mL)** | **Continuation**  **p < 0.001** | **Reduction**  **p < 0.001** | **Intensification**  **p < 0.001** | **Discontinuation**  p = 0.103 | **Active Disease**  p = 0.408 |
| --- | --- | --- | --- | --- | --- | --- |
| Q1 (21) | 0.18–5.4 | 2 (10%) | 0 (0%) | 15 (71.4%) | 4 (19%) | 8 (38%) |
| Q2 (21) | 5.9–12.9 | 15 (71%) | 0 (0%) | 5 (23.8%) | 1 (5%) | 5 (23%) |
| Q3 (20) | 13.0–20.1 | 12 (60%) | 5 (25%) | 2 (10.0%) | 1 (5%) | 3 (15%) |
| Q4 (20) | 20.3–52.7 | 10 (50%) | 10 (50%) | 0 (0%) | 0 (0%) | 4 (20%) |

Values are presented as n (%). Statistical analysis consisted of Fisher's exact test; p values < 0.05 are bold.

**Supplemental Table 4: Clinical outcomes stratified by IFX clearance quartiles**

| **Quartile (sample n)** | **Clearance range (L/day)** | **Continuation**  p = 0.128 | **Reduction**  **p = 0.030** | **Intensification**  **p = 0.002** | **Discontinuation**  p = 0.206 | **Active Disease**  p = 0.124 |
| --- | --- | --- | --- | --- | --- | --- |
| Q1 (21) | 0.044–0.224 | 14 (67%) | 6 (29%) | 1 (5%) | 0 (0%) | 4 (19%) |
| Q2 (21) | 0.224–0.273 | 11(52%) | 6 (29%) | 3 (14%) | 1 (5%) | 3 (14%) |
| Q3 (20) | 0.273–0.344 | 7 (35%) | 3 (15%) | 8 (40%) | 2 (10%) | 4 (20%) |
| Q4 (20) | 0.352–0.923 | 7 (35%) | 0 (0%) | 10 (50%) | 3 (15%) | 9 (45%) |

Values are presented as n (%). Statistical analysis consisted of Fisher's exact test; p values < 0.05 are bold.

**Supplementary Table 5. Association between PK predictors and IFX therapy outcomes (intensification, discontinuation, or reduction), based on predictors identified as significant in univariate logistic regression (Table 2).**

| **Outcome** | **Intensification** | | | **Discontinuation** | | | **Continuation** | | | **Reduction** | | | **Intensification** | | |
| --- | --- | --- | --- | --- | --- | --- | --- | --- | --- | --- | --- | --- | --- | --- | --- |
| **Variables in model** | OR | 95% CI | p value | OR | 95% CI | p value | OR | 95% CI | p value | OR | 95% CI | p value | OR | 95% CI | p value |
| PK Predictor* | **26.7** | **(4.07, 260.8)** | **0.002** | 19.7 | (0.73, 1399.9) | 0.09 **.** | 1.72 | (0.56, 5.42) | 0.346 | **13.9** | **(2.73, 101.9)** | **0.003** | **12.6** | **(2.45, 99.1)** | **0.006** |
| Antibody Positive | 1.65 | (0.14, 23.8) | 0.69 | 1.38 | (<0.001, 3626.7) | 0.92 | **0.06** | **(0.002, 0.62)** | **0.04** | ~ 0 | (NA, ∞) | 0.99 | 6.59 | (0.90, 74.8) | 0.08 **.** |
| Albumin < 4 g/dL | 0.50 | (0.09, 2.43) | 0.40 | 1.50 | (0.04, 97.1) | 0.82 | 1.60 | (0.50, 5.46) | 0.44 | 1.01 | (0.19, 5.68) | 0.99 | 0.44 | (0.08, 2.13) | 0.32 |
| Active Disease (PGA > 0) | 1.27 | (0.23, 6.46) | 0.77 | 60.1 | (1.72, 9096.6) | 0.05 **.** | 0.33 | (0.08, 1.24) | 0.11 | 0.48 | (0.02, 4.23) | 0.55 | 1.84 | (0.37, 9.33) | 0.45 |
| Female Sex | 2.09 | (0.47, 10.3) | 0.34 | ~ 0 | (NA, ∞) | 1.00 | 1.73 | (0.59, 5.36) | 0.33 | 0.33 | (0.05, 1.73) | 0.22 | 2.70 | (0.59, 14.9) | 0.22 |
| Age > 40 Years | 0.86 | (0.19, 3.95) | 0.85 | 2.23 | (0.03, 561.9) | 0.72 | 1.33 | (0.43, 4.28) | 0.62 | 0.36 | (0.06, 1.87) | 0.24 | 0.75 | (0.16, 3.40) | 0.70 |
| Crohn’s Disease | 0.68 | (0.04, 8.68) | 0.77 | 0.48 | (<0.001, 589.8) | 0.86 | 2.20 | (0.35, 16.1) | 0.41 | 0.80 | (0.06, 9.73) | 0.86 | 1.32 | (0.09, 15.8) | 0.83 |
| Montreal Location L3 | 1.82 | (0.32, 12.3) | 0.51 | 0.22 | (<0.001, 22.3) | 0.52 | 0.99 | (0.23, 4.18) | 0.99 | 0.44 | (0.05, 3.62) | 0.44 | 1.17 | (0.22, 6.93) | 0.86 |
| Montreal Behavior B2/B3 | **10.3** | **(1.49, 118.6)** | **0.03** | 1.94 | (0.01, 3615.9) | 0.83 | 0.22 | (0.04, 1.02) | 0.06 **.** | 2.04 | (0.21, 22.2) | 0.54 | **7.92** | **(1.36, 71.3)** | **0.03** |
| Perianal Disease | 0.61 | (0.10, 3.30) | 0.57 | 0.12 | (<0.001, 13.8) | 0.40 | 3.69 | (0.82, 19.4) | 0.10 | 0.35 | (0.01, 3.84) | 0.43 | 0.31 | (0.05, 1.70) | 0.20 |
| Concomitant Medication | 0.63 | (0.14, 2.56) | 0.52 | 14.9 | (0.54, 2012.7) | 0.17 | 0.88 | (0.30, 2.58) | 0.82 | 1.62 | (0.32, 8.44) | 0.56 | 0.50 | (0.11, 1.93) | 0.33 |
| Weight < 35 kg | ~ 0 | (NA, ∞) | 0.99 | ~ 0 | (NA, ∞) | 1.00 | ~ 0 | (NA, ∞) | 0.99 | ~ 0 | (NA, ∞) | 1.00 | ~ 0 | (NA, ∞) | 0.99 |
| **PK Predictor (cutoff)*** | Forecasted Trough (< 5 μg/mL) | | | | | | (> 10 μg/mL) | | | (> 15 μg/mL) | | | Clearance (> 0.294 L/day) | | |

Values shown are odds ratios (OR), 95% confidence intervals (CI), and p-values. (*) Indicates primary PK predictor (forecasted trough <5 µg/mL, > 10 µg/mL, >15 µg/mL, or clearance >0.294 L/day, as specified). ORs and p-values that are significant (p<0.05) are bold. (.) indicates p-values between 0.05 and 0.10, suggesting a trend toward significance. IFX: infliximab or biosimilars; PK: pharmacokinetics.

**Supplementary Figure 1: Receiver operating characteristic (ROC) curves assessing the predictive performance of PK predictors on IFX therapy outcomes and active disease status in patients with IBD.**

ROC curves illustrate the ability of PK parameters -forecasted IFX trough concentrations (µg/mL, blue line) and clearance (L/day, green line) -to predict clinical outcomes: a) IFX therapy intensification, b) IFX therapy continuation, c) IFX therapy discontinuation, d) IFX therapy reduction, and e) active disease (PGA>0). Area under the curve (AUC) values are presented in each panel, with higher AUCs indicating greater predictive accuracy. Corresponding optimal cutoff thresholds, sensitivity, specificity, Youden indices, and statistical significance (p values) derived are summarized in the inset table. Statistically significant results (p < 0.05) are shown in bold. A dot (.) indicates p-values between 0.05 and 0.10, suggesting a trend toward significance. Abbreviations: Sens: Sensitivity; Spec: Specificity; IFX: infliximab; PK: pharmacokinetic; PGA: Physician Global Assessment.
