# Supplementary figures and images for: Impact of Precision-Guided Dosing on Clinical Decision-Making and Health Care Utilization in Inflammatory Bowel Disease: A Retrospective Pretest/Posttest Real-World Study
Source: Crohns Colitis 360. 2025 Jun 24;7(3):otaf044. doi: 10.1093/crocol/otaf044 (PMC12305531; doi:10.1093/crocol/otaf044)

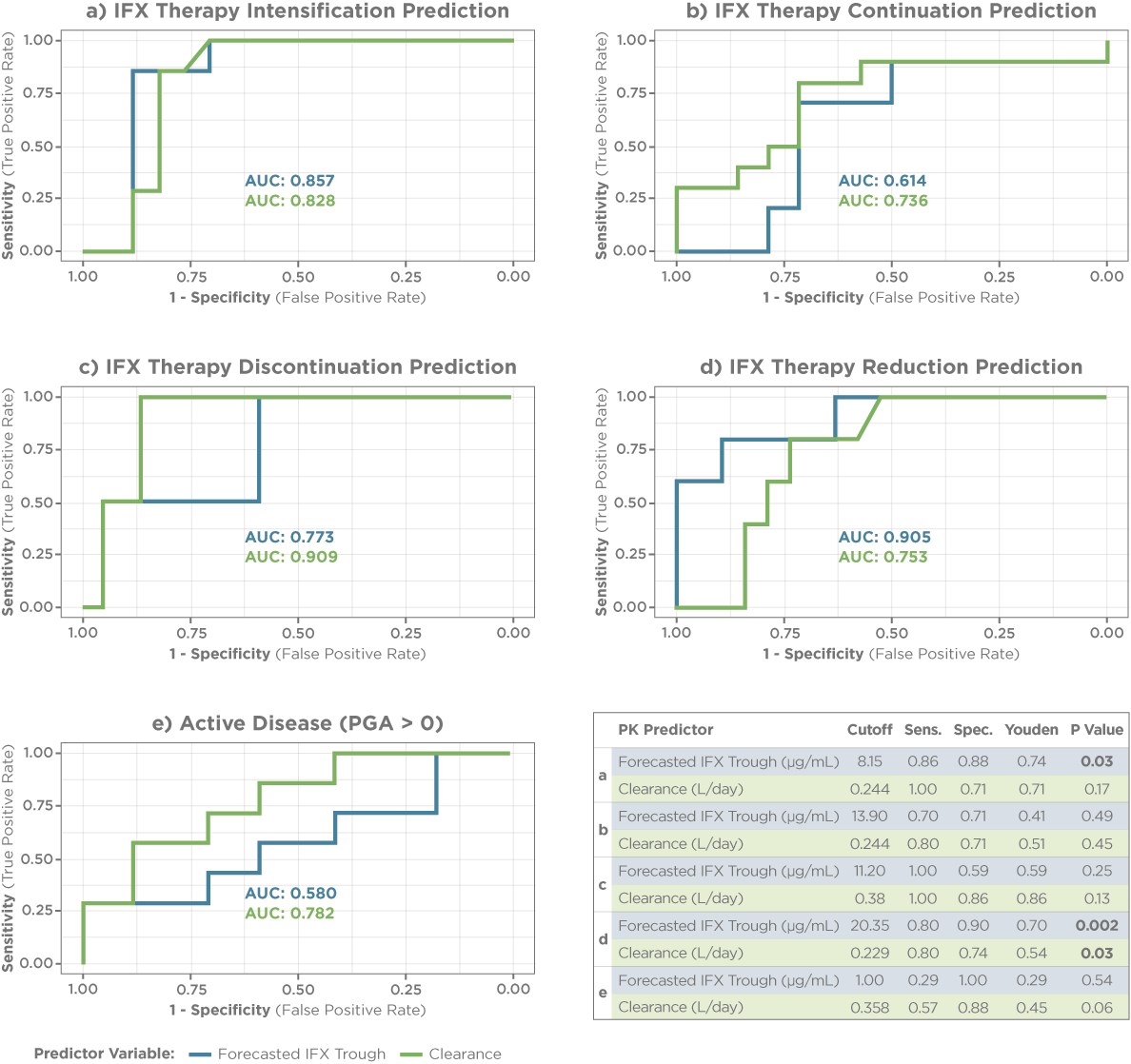

Supplement: otaf044_suppl_Supplementary_Figure_S1 [file otaf044_suppl_supplementary_figure_s1.jpeg]
